# The Effects of Reduced Graphene Oxide Flakes in the Dielectric on Electrical Discharge Machining

**DOI:** 10.3390/nano9030335

**Published:** 2019-03-02

**Authors:** Rafał Świercz, Dorota Oniszczuk-Świercz

**Affiliations:** Institute of Manufacturing Technology, Warsaw University of Technology, 00-661 Warsaw, Poland; doo@meil.pw.edu.pl

**Keywords:** EDM, electrical discharge machining, RGO, reduced graphene oxide, powder mixed, surface roughness, recast layer

## Abstract

Electrical discharge machining (EDM) is a nonconventional technology that is frequently used in manufacturing for difficult-to-cut conductive materials. Drawbacks to using EDM include the resulting surface roughness and integrity. One of the recent innovations for improving surface integrity with EDM is the use of a powder mixed dielectric. The aim of this study is to analyze the influence of having reduced graphene oxide (RGO) in the dielectric on the ionization of the plasma channel and the dispersion of electrical discharges. The main goal is to improve the surface integrity of the tool steel 55NiCrMoV7 during finishing machining. To achieve this goal, an experimental investigation was carried out to establish the smallest possible values of discharge current and pulse time at which it is possible to initiate an electric discharge, which causes material removal. Next, the effect of the direction of the electric discharges (electrode polarity) and the concentration (percentage) of RGO in the dielectric on surface integrity was investigated. The results of this experiment indicate that during EDM with RGO, the discharges are dispersed on the RGO flakes. This leads to a multiplication of the discharges during a single pulse, and this strongly affects the surface integrity. The obtained results indicate that it is possible to reduce surface roughness and thickness of the recast layer by approximately 2.5 times compared with conventional EDM.

## 1. Introduction

Electrical discharge machining (EDM) is frequently used to manufacture difficult-to-cut conductive materials. In the EDM process, the material is removed from the workpiece through a series of electrical discharges that occur in the gap between electrodes immersed in the dielectric. The removal mechanism of the material in EDM is mainly the result of the electrical discharge causing the melting and evaporation of the local surface layers of both the workpiece and the electrode. Collapsing the plasma channel at the end of the discharge induces high-pressure waves that wash over the molten and evaporated metal. The overlapping traces of the individual discharges generate a specific surface topography. The heat flux transferred to the workpiece during the discharges changes the microstructure of the external surface layers. Analyses of the microstructure of tool steel after EDM show layers that typically result from the process: a recast layer, a heat-affected zone, and a tempered layer, whose properties are different from the core material. The industrial application of EDM requires the evaluation of the properties of the surface integrity of the manufactured parts. The main factors that influence the surface properties after EDM can be split into the parameters of the electrical discharge (discharge current, discharge voltage, pulse time, duty factor) and processing conditions, electrode material, workpiece material, and type of the dielectric [1,2,3,4]. To produce high-precision components with low surface roughness and wear, many works have focused on improving surface integrity after EDM. There are two major fields of study for this purpose: using additional technologies, such as electrochemical machining [5,6], applying coatings [7,8,9,10], and nonconventional finishing [11,12,13,14,15,16], or improving the EDM process [17,18,19,20,21,22,23,24]. Since adding technologies to the manufacturing process increases costs, there have been several recent innovations for improving the surface integrity during the EDM process. One such method is the use of a powder mixed dielectric (PM). 

The physics of material removal in powder mixed electrical discharge machining (PMEDM) are determined by the properties of the medium in which the electrical discharges occur. The dielectric fills the gap between electrodes and acts as a spark conductor. Appropriate dielectric properties ensure stable discharges, cool the workpiece and electrode surface, and flush debris particles from the gap and quench the plasma channel [25]. Effective removing of debris from the gap ensures stabile discharges and repeatable effect of the process. Size of the spherical debris depends on the used discharge energy and is in the range of 1 nm to 10 µm. Murray et al. [26] show that the presence of debris in the gap changes local electric-field strength. The authors indicate that it is possible to predict the electric-field strength as a function of debris concentration in the gap. For short time intervals between discharges not all debris may be removed from the gap. This provides to increases the probability of another discharge being in the same place. Furthermore, the ineffective removal of the products of erosion leads to a short circuit. Using EDM with additional conductive particles in the dielectric contamination facilitates ignition process with increases gap size. This allows better flushing of the gap and removing the debris. Wang et al. [27] analyzed the breakdown process of a dielectric and the growth of plasma channels through single-pulse discharges. Their research indicates that the discharge channel is compressed by the bridge of conductive particles stabilizing the narrow plasma channel. Research carried out by [28,29,30] confirms that ionization of the plasma channel with conductive particles in the dielectric occurs in the larger gap relative to a pure dielectric, and this significantly affects the EDM process. The surface integrity after EDM can vary significantly, depending on the type of particles used in the dielectric. Marashi [31] et al. analyzed the influence of Ti nanopowder in the dielectric on the surface integrity of AISI D2 steel. Their research shows that using Ti nanopowder in the dielectric improves the material removal rate (MRR) and surface roughness. Nguyen et al. [32] concluded that using Ti powder in the dielectric not only reduces surface roughness but also considerably increases the microhardness of the machined surface layers. Amorim et al. [33] investigated the influence of different sizes of fine Mo powder particles in the dielectric on the surface integrity. Their presented results reveal that it is possible to produce a Mo-enriched layer with increased hardness in a tool steel workpiece. Toshimitsu et al. [34] used chromium powder in the dielectric to achieve a corrosion-resistant coated layer. Sharad et al. [35] investigated the influence of boron carbide abrasive particles in the dielectric while machining grade-5 Ti alloy. Their results show that the concentration of powder in the dielectric has a strong influence on the MRR and tool wear rate (TWR). TWR is reduced and MRR increases at higher abrasive concentration levels of 10 g/L. Kumar et al. [36] used Al_2_O_3_ nanopowder in deionized water to improve the surface integrity of Inconel 825. The presented results indicate that microcracks can be reduced drastically by using power mixed EDM compared with conventional EDM. Research presented by Patel et al. [37] of the influence of EDM particles with aluminum oxide (Al_2_O_3_) when machining Inconel 718 indicates that discharge parameters have a significant effect on machining efficiency. The concentration of the powder in the dielectric influences the surface integrity. Talla et al. [38] proposed the use of graphite powder in the dielectric to improve the surface integrity of Inconel 625 after EDM. Their results indicate a significant reduction in surface roughness, crack density, and white layer thickness using the PMEDM process. Research carried out by Mohanty et al. [39] indicates that a hard and solid-lubricating layer over Ti6Al4V can be obtained using tungsten disulfide in the dielectric deionized water. Al-Khazraji et al. [40] pointed out that the electrode material used in PMEDM with SiC in the dielectric has a strong influence on the process. Hourmand et al. [41] reported that adding aluminum nanopowder to the dielectric during EDM of an Al–Mg_2_Si composite decreases the spark energy, which leads to fewer microstructural changes in the material. Bains et al. [42] aimed to improve PMEDM by magnetic field-assisted finishing. The results show that while machining in a magnetic field coupled with a higher spark, it is possible to reduce the thickness of the recast layer, and this is accompanied by a significant effect on the MRR. 

The published literature indicates that a few studies have reported the influence of nanopowder in the dielectric during EDM, but the use of RGO flakes in the dielectric has not been sufficiently described. Due to the properties of RGO flakes (high electrical and thermal conductivity) [43,44,45,46,47], there is a different course of electric discharge and the final process of heat dissipation in the interelectrode gap relative to the process in kerosene. Facilitating the initiation of an electrical discharge (with an increased interelectrode gap in relation to the standard thickness) results in stable electrical discharges with reduced energy. 

The topic of this article is the influence of RGO in the dielectric on the ionization of the plasma channel and the dispersion of electrical discharges. The main goal of the study is to improve the surface integrity of the tool steel 55NiCrMoV7 during finishing machining. To achieve this goal, an experimental investigation was carried out to examine three cases. In the first stage, an experiment was carried out with the goal of establishing the values of electrical parameters: discharge current and pulse time (as small as possible) at which it is possible to initiate an electric discharge that causes material removal. The second goal is to investigate the influence of the direction of the electric discharge (electrode polarity) on the surface layers and roughness. In the last stage, the influence of the percentage of RGO in the dielectric on the surface integrity was investigated.

## 2. Materials and Methods 

**Figure 1:**
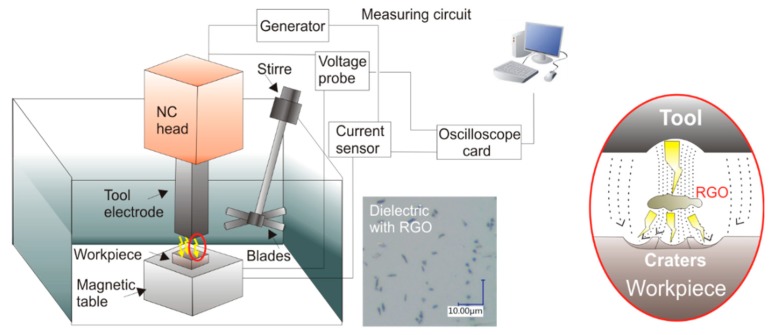
Schematic illustration of experimental setup.

The experimental research was performed using a Charmilles Form 2 EDM machine (GF Solutions, Geneva, Switzerland). A copper electrode with a cross-sectional area of 12 × 12 mm was used. Heat-treated samples of the tool steel 55NiCrMoV7 (55 HRC) had dimensions of 10 × 10 × 3 mm. This material is widely used for forging dies, die inserts, and dies for hydraulic and mechanical presses. Figure 1 shows the schematic diagram of the setup. The dielectric was the commercial EDM fluid 108 MP-SE 60 with different concentrations of reduced graphene oxide (RGO). To prevent the RGO in the dielectric from concentrating in one place, the working fluid was stirred with a rotating screw during EDM. The average size of the RGO was about 2 µm. All samples used in the tests were ground prior to EDM to achieve the same surface roughness properties.

The main objective of the study is to investigate the influence of RGO in the dielectric on surface integrity during finishing machining. The geometric structure of the surface was measured made using a Taylor Hobson FORM TALYSURF Series 2 scanning profilometer (Taylor Hobson, Leicester, United Kingdom). The roughness parameter was measured on a surface area of 2 × 2 mm with a discretization step (10 μm) in the *X*-axis and *Y*-axis. 

To characterize the surface topography after EDM, the following 3D surface roughness parameters were used:

- *Sa*: arithmetic mean of the deviations from the mean (average value of the absolute heights over the entire surface).
- *Sdq*: mean quadratic slope of the surface.
- *Ssc*: arithmetic mean curvature to the top—this parameter represents whether the mean form of the peaks is pointed or rounded according to the mean value of the curvature of the surface at these points.
- *Sds*: density of the top (the number of summits of a unit sampling area), which relies on the definition of the eight nearest-neighbor summits, where a peak is defined if it is higher than its eight nearest neighbors.

Metallographic surface structure studies were performed using a Nikon Eclipse LV 150 optical microscope (Nikon, Tokyo, Japan) coupled to a NIS-Elements BR 3.0 image analyzer (Nikon). Specimens were encased in resin and then machined with grinding and polishing. Micro-etching was performed with nital (5%) to reveal the microstructure of the material.

The RGO in the dielectric was observed using a Keyence VHX-6000 (Keyence, Osaka, Japan) digital microscope.

**Figure 2:**
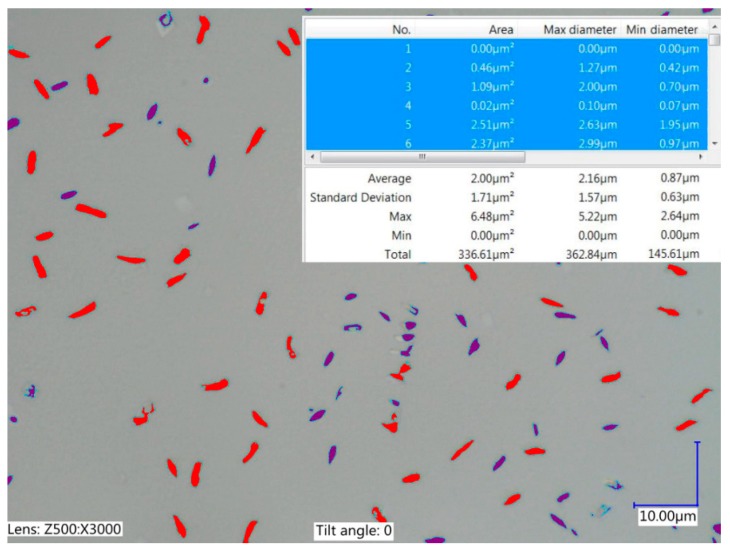
Reduced graphene oxide in the dielectric: calculating the average area of RGO.

The RGO was developed in the Graphene Laboratory of the Warsaw University of Technology. The RGO used in the study had an average area of 2 µm^2^ (Figure 2). Its properties and production method were investigated and presented by Stobiński et al. [48].

The physics of material removal in EDM depend on current and voltage parameters and the properties of the dielectric in which the electrical discharges occur. Adding RGO flakes to the dielectric changes the processes of initiation and propagation of discharges. First, a test was conducted to investigate a range of stability discharges for different values of discharge currents and pulse times and to assess the RGO percentage in the dielectric during finishing machining. The current and voltage waveforms during the EDM process were measured using a National Instruments NI5133 oscilloscope card (National Instruments, Austin, TX, USA). An application was developed in the LabView environment that enabled controlling the oscilloscope card function. The current was measured indirectly by determining the voltage drop on the non-inductive current sensor. The maximum value of the voltage drop for the set current values did not exceed 3V, so the signal was fed directly to the oscilloscope card. The voltage during the electric discharge was measured with a Tektronix probe (Tektronix UK Ltd., Berkshire, UK). The sampling rate was 100 MS/s, 2-Channel registration. The obtained data were analyzed in DIAdem (National Instruments).

**Figure 3:**
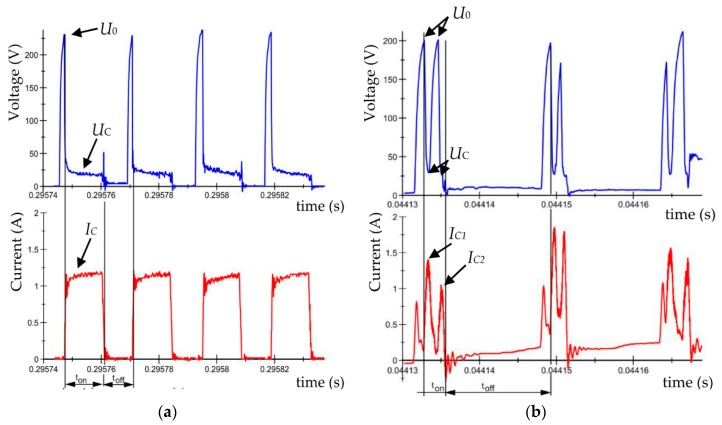
Recorded voltage and current waveforms: EDM fluid dielectric (**a**) *U*_0_ = 225 V, *U* = 25 V, *I* = 1.2 A, *t*_on_ =10 µs, *t*_off_ = 8 µs, pure dielectric; and (**b**) *U*_0_ = 200 V, *U_C_* = 25 V, *I* = 1.5 A, *t*_on_ = 3 μs, t_off_ = 13 μs, EDM fluid with 0.1% RGO.

Exemplary current and voltage waveforms of EDM in the pure dielectric and the dielectric with RGO are shown in Figure 3. 

The workpiece was machined at the moment when the supply voltage *U*_o_ dropped to the discharge voltage *U*_C_ and there was an increase in the discharge current *I*c during the pulse *t*_on_. The electrical voltage supplied by the generator to the electrodes immersed in the dielectric generated a time-varying electric field (110 V/m). The initiation of the electrical discharge caused a high voltage, which overcame the dielectric breakdown strength. Emitted electrons collided with atoms, leading to the local ionization of the dielectric and the generation of the plasma channel. The current flowing through the plasma channel caused the melting and evaporation of the workpiece and working electrode. Around the plasma channel, a gas bubble formed that was filled with compounds and ions of molten metal. At the end of discharge, the voltage and current dropped. The plasma channel and implosive gas bubble collapsed and expelled erosion products into the gap. Some of the material that was not removed from the crater resolidified on the surface. The conditions in the gap then stabilized. 

Analyses of the current and voltage waveforms in the dielectric with RGO show significant differences in the initiation and shape of the discharge waveform. In the first stage with rising voltage, the current increased due to the presence of conductive particles in the dielectric that locally decrease the dielectric breakdown strength. Next, the current dropped, and the voltage rose to the set supply voltage *U*_0_. Finally, high voltage overcame the dielectric breakdown strength and dropped to the discharge voltage *U*_C._ At the same time, the current rose to the discharge current *I*c_1_. Due to the presence of RGO flakes in the dielectric, for one pulse, more than one discharge occurred (in the other place that discharges occur). The multi-discharge effect was caused by the dispersion of the discharge energy. RGO flakes facilitated the bridging effect and minimized the insulating strength of the dielectric. 

## 3. Results and Discussion

### 3.1. Analysis of Influence of RGO in the Dielectric on the Discharge Propagation.

**Figure 4:**
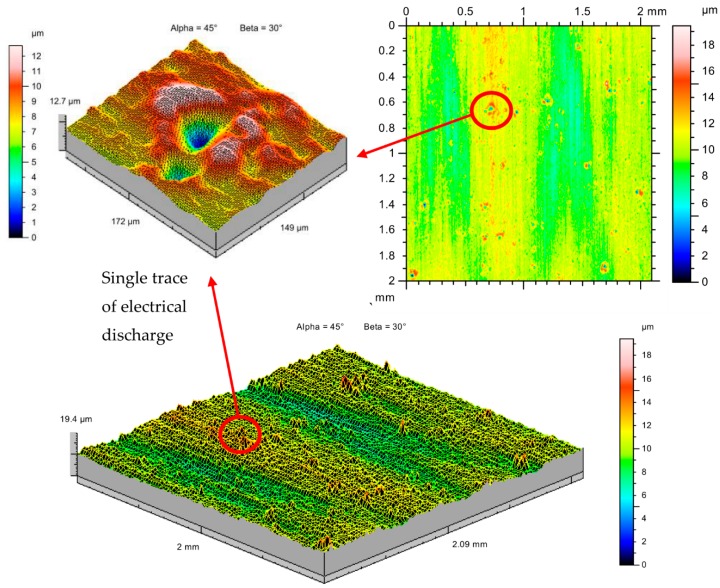
The surface texture of the tool steel 55NiCrMoV7 after powder mixed electrical discharge machining (PMEDM) in dielectric with 2% RGO; *U_c_* = 25 V, *I* = 3 A, *t*_on_ = 15 µs, *t*_off_ = 10 µs, manufacturing time = 60 s.

The preliminary tests show that when RGO flakes at a concentration above 1% are in the dielectric, the EDM parameters—current *I* (0.5–3 A) and pulse t_on_ (5–15 μs)—are not uniform, and the effective electric discharges lead to erosion of the material. The recorded voltage and current signals (using the developed measuring circuit) show the occurrence of the dielectric breakdown and the emission of electrons, but there are only single traces of discharges on the material. Discharge energy is scattered on the reduced graphene oxide flakes. The single craters on the manufacturing surface in Figure 4 indicate that for the analyzed parameters, there is only electron dispersion on RGO flakes without discharges. The occurrence of local craters is likely the result of the inhomogeneity of the distribution of RGO flakes in the dielectric. There was not a stable breakdown of dielectric and ionization of the plasma channel. Analysis of the topography of the surface shows that it is characteristic of the grinding process (visible directional structure). The analyzed surface did not change its properties as a result of the EDM process. 

**Figure 5:**
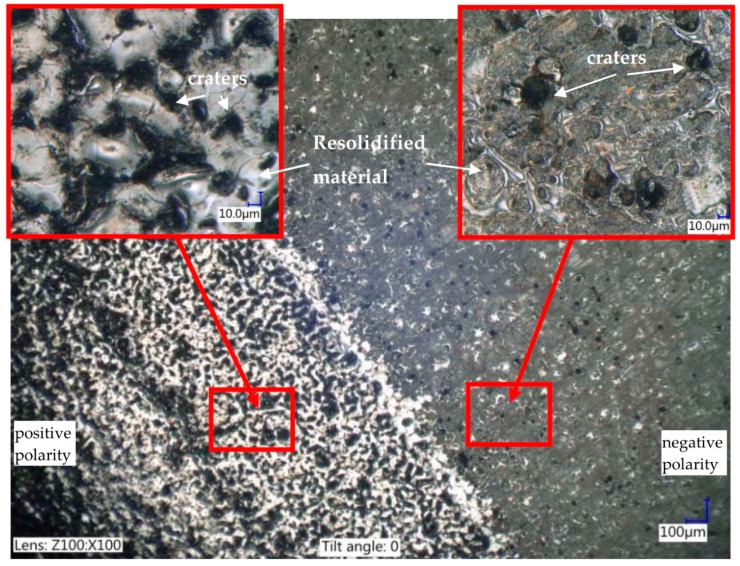
The surface texture of the tool steel 55NiCrMoV7 after PMEDM in the dielectric with 2% RGO, positive polarity, and negative polarity; manufacturing time = 60 s.

The next stage of this research was the analysis of different polarities of the electrode during PMEDM. In most cases of skinning, the electrode of an EDM tool has a positive polarity. Choosing this type of polarity for the manufacturing process decreases tool wear when using discharges with a long duration. This is a result of the deposited carbon layer on the tool electrode (anode), and the carbon deposition is caused by the thermal dissociation of a carbon dielectric. If the finishing machining uses a short pulse time, the deposition of carbon is much less. Using negative polarity for an electrode decreases the discharge energy transferred to the workpiece. Tests conducted using positive and negative polarity of the electrode during EDM with RGO flakes in the dielectric show a significant difference in surface topography (Figure 5). Only single craters are observed for PMEDM using a negative electrode polarity, low discharge currents, and short time pulses—*Ic* = 3 A and t_on_ = 10 µs, respectively—and 2% RGO in the dielectric on the manufacturing surface. The discharge energy scatters over the RGO flakes, and the single craters are the result of a local breakdown of the dielectric trough’s electrically charged RGO flakes. Using positive polarity for the electrode (for the same PMEDM conditions) causes the generation of stable discharges through RGO as a result of the uniformly manufactured surface. 

In the first stage of the conducted research, the measurement circuit was developed to determine the current-voltage characteristics of the generator machines. A primary test was performed to investigate a range of the stability discharges for different values of the discharge current, pulse time, for different % RGO in the dielectric. According to conducted preliminary research and literature review, the range of investigated parameters for finishing EDM machining was established. The experiment was carried out with the goal of determining the values of electrical parameters: discharge current and pulse time (as small as possible) at which it is possible to initiate an electric discharge that causes material removal. Purpose of the work was to analyses of the possibility of using RGO flakes to improve surface quality during finishing machining. The preliminary experimental studies indicate a stable propagation of electrical discharges in the dielectric containing RGO flakes at concentrations that do not exceed 1% (for the finishing range of EDM parameters, the discharge current, pulse time, and discharge voltage are *I* = 1–3 A, *t*_on_ = 5–15 μs, *U_c_* = 25 V). After analyzing these results, the following machining conditions were selected (Table 1). 

### 3.2. Analysis of Surface Topography

**Figure 6:**
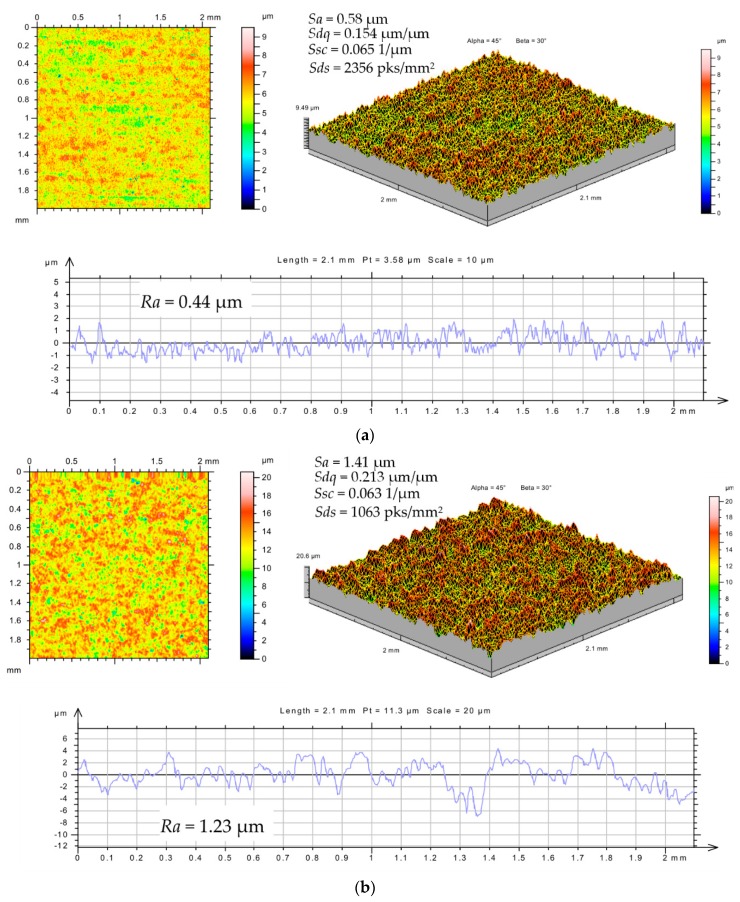
The surface texture of the tool steel 55NiCrMoV7 after EDM (*U_c_* = 25 V, *I* = 1 A, *t*_on_ = 5 µs, *t*_off_ = 5 µs) with positive polarity (**a**) in dielectric with 0.1% RGO and (**b**) in the pure dielectric.

The surface topography after EDM is determined by overlapping traces of individual electrical discharges and has a point random character. Both the ordinates and the local elevations for the measured samples have normal distributions, leading to unfavorable properties of the surface carriers. Depending on the analyzed concentration of RGO in the dielectric and the applied polarity, there are significant differences in the structure of the surface topography (Figure 6).

One of the goals of the research is to investigate the influence of the discharge current, discharge time, and RGO concentration (%) in the dielectric on the surface topography during finishing machining. Having completed the preliminary tests to select the range of stable discharges for different polarities, the next experimental test was carried out. Three pairs of each samples have been manufactured to compare the repeatability of the process. Table 2 synthesizes the measurement results of the surface topography parameters of tool steel 55NiCrMoV7 samples. Included in Table 2 are the parameters that provide stable, effective electric discharges, as well as the smallest achievable values of discharge current and pulse time that cause erosion of the manufacturing material with RGO in the dielectric.

Analysis of the obtained results from experimental studies shows that there are significant differences in surface topography depending on the percentage concentration of RGO in the dielectric and the type of polarity used. The arithmetic mean of the deviations from the mean *Sa* range from 0.58 to 1.56 μm when machining with positive polarity and from 0.44 to 1.13 μm with negative polarity. These values correspond to the roughness in finishing machining. There is a significant decrease in roughness *Sa* when manufacturing with 0.1% RGO flakes in the dielectric. 

The mean quadratic slope of the surface (*Sdq*) changed from 0.154 to 0.213 µm/µm for positive polarity and from 0.067 to 0.114 µm/µm for negative polarity. The results of the tests show that applying negative polarity in each analyzed case results in a much smaller value of the quadratic slope of the surface compared with the application of positive polarity.

The arithmetic mean curvature to the top of the peaks (*Ssc*) varies from 0.063 to 0.067 1/µm and 0035 to 0.042 1/µm for positive and negative polarity, respectively. For the negative polarity, a smaller value of rounding the vertices was obtained. The *Sds* and *Ssc* values significantly affect the abrasive wear of the surface, the application of coatings, and reflectivity. With a smaller rounding radius of the vertices, the top has a sharp edge, which leads to an increase in the coefficient of friction. This enhances the adhesive properties of the surface.

**Figure 7:**
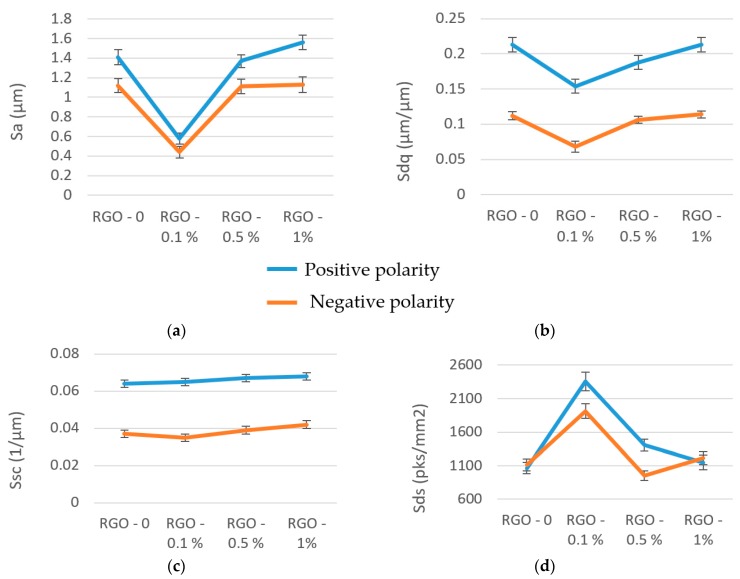
The surface roughness parameters after PMEDM of the tool steel 55NiCrMoV7 with positive and negative polarity: (**a**) *Sa*; (**b**) *Sdq*; (**c**) *Ssc*; (**d**) *Sds*.

The surface roughness parameter that describes the average height of roughness, *Sa* (Figure 7a), primarily depends on the % RGO in the dielectric. The obtained *Sa* value with 0.1% RGO is almost 2.5 times smaller than that with the pure dielectric. Similar roughness *Sa* values are observed for RGO concentrations of 0.5, 1, and 0% (i.e., pure dielectric). However, PMEDM with 0.5 and 1% RGO result in larger values of discharge current and pulse time compared with the pure dielectric. These results indicate that when performing PMEDM with RGO, there is a dispersion of discharges on the RGO flakes during a single pulse. This causes the multiplied discharges with lower energies to reach the surface of the workpiece.

The shape of the roughness vertices can be described by the arithmetic mean curvature to the top (*Ssc*), the density of the top (*Sds*), and the quadratic slope of the surface (*Sdq*). The values of the *Ssc* and *Sdq* parameters depend mainly on the polarity (Figure 7b,c). For positive polarity, the higher discharge energy is transferred to the workpiece, leading to the melting and evaporation of the material. This leads to the crater (generated by the electrical discharges with positive polarity) having the largest diameter and height and rounded vertices. 

The density of the top (*Sds*) differs quite significantly between PMEDM with negative and positive polarity for 0.1% RGO in the dielectric (Figure 7d). This is because the *Sds* parameter depends on both the height of the roughness and the distance between summits. The *Sds* parameter represents the number of summits in a sampling area and indirectly defines the distance between adjacent summits. A higher value of *Sds* means that there are more summits for the same area size. The changed distance between adjacent summits with similar values of roughness/peak height parameters can be an indicator that the crater has a similar height but different diameter.

For EDM with additional powder the electrical resistivity of the dielectric decreases. RGO flakes in the dielectric reduced breakdown voltage. This result of bridge formation. The conductive flakes are polarized in the electric field. The gap is larger than the conventional EDM. This provides better flushing and removed debris. Razing the gap size leads to decreases the heat flux and volume of material removed in discharge. Discharge dispersion reduced the emergence of surface ridges. Generated craters are shallow with lower borders. Depends on the used particle properties (size, electric conductivity, thermal conductivity) for the same EDM parameters different effects can be obtained. Presented results indicate similar trends to result presented by Jahan et al. [49] with the used of graphite powder. Concertation of additional particles has a strong influence on the surface roughness [30]. Conducting research indicate that for using RGO in the dielectric increase the concentration (from 0.1% to 0.5% and 1%) required to increase discharge energy to local melting the material. An explanation of this effect can be found in RGO properties. The high electrical conductivity of RGO cause dispersion of electrons on the RGO flakes. For the lower value of discharge current and time pulse, *I* = 1 A, *t*_on_ = 5 µs and RGO concertation 0.5% and 1% energy which is transferred to the surface is not enough to melt and evaporation of material.

### 3.3. Analysis of the Surface Layer

**Figure 8:**
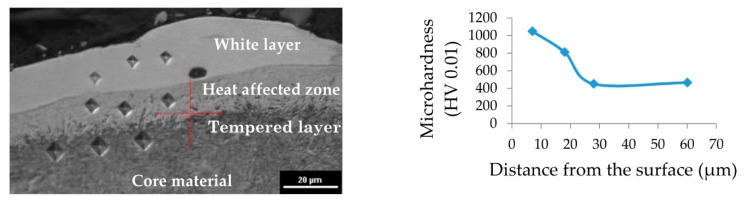
Metallographic structure of tool steel 55NiCrMoV7.

**Figure 9:**
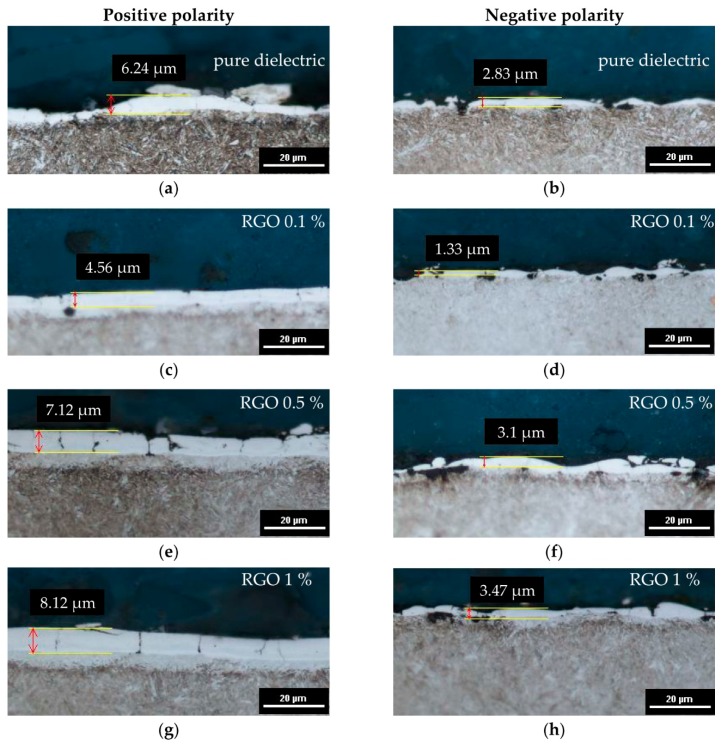
Metallographic structure of tool steel 55NiCrMoV7 after PMEDM: (**a**,**b**) *Uc* = 25 V, *I* = 1 A, *t*_on_ = 5 µs, *t*_off_ = 5 µs, dielectric without RGO; (**c**,**d**) *Uc* = 25 V, *I* = 1 A, *t*_on_ = 5 µs, *t*_off_ = 5 µs; RGO = 0.1%; (**e**,**f**) *Uc* = 25 V, *I* = 2 A, *t*_on_ = 8 µs, *t*_off_ = 5 µs, RGO = 0.5%; (**g**,**h**) *Uc* = 25 V, *I* = 2 A, *t*_on_ = 8 µs, *t*_off_ = 5 µs, RGO = 1%.

EDM is a nonconventional technology in that material is removed from the workpiece as a result of a series of discharges. During a single electrical discharge, the temperature inside the plasma channel is estimated to be between 6000 and 7000 K [50]. Heat flux causes melting and evaporation of both the workpiece and the working electrode. At the end of the discharge, the plasma channel and surrounding implosive gas bubble collapse and expel liquid metal into the gap. Some of the molten material which was not removed from the crater resolidifies on the surface. Due to the melting and evaporating of material during electrical discharges, the resolidified material may have the chemical composition of the workpiece and electrode material. Analysis of the metallographic surface of the tool steel 55NiCrMoV7 after EDM reveals the appearance of characteristic variations in the surface layers (Figure 8). The thickness of the observed layer depended on the PMEDM parameters (Figure 9). 

Under the recast layer (commonly called the white layer—WL) in the metallographic structure, a heat-affected zone (HAZ) is observed. This layer is visible as a bright structure directly under the white layer. The HAZ and white layer are characterized by increased microhardness relative to the core material. The bottom-most layer is the tempered layer, which is visible as a dark streak immediately below the HAZ. For the investigated range of parameters (Table 2), recast layers were observed for all samples. 

The white layer that appears after PMEDM is characterized by large variations in thickness when using negative polarity and when using the pure dielectric (Figure 9a,b,d,f,h). The thickness of the white layer is non-homogeneous and dependent on the machining parameters used in experiments. The discontinuity of the recast layer is caused by the random occurrence of electrical discharge and a smaller volume of material being removed during a single discharge in negative polarity. With RGO in the dielectric and positive polarity, the thickness of the recast layer is uniform (Figure 9c,e,g). This is the result of more stable electrical discharges with less energy in the dielectric containing RGO. The minimal thickness of the white layer observed for PMEDM with an RGO of 0.1% and negative polarity is WL = 1.33 µm, which is two times smaller than the thickness of the white layer achieved in the pure dielectric. For PMEDM with RGO concentrations of 0.5% and 1%, the white layer is slightly thicker than with the pure dielectric. This is the result of using a 2-fold higher discharge current. 

Analyses of the metallographic images indicate that the using of RGO in the dielectric leads to a more uniform distribution of the recast layer on the surface. An explanation of this effect can be found in RGO properties. In conventional EDM molten material which is not removed from workpiece after discharges are rapidly cooled by the dielectric. In the case of EDM with RGO in the dielectric during the discharge RGO flakes stores the heat energy. After discharge RGO flakes give back this energy to the dielectric. This phenomenon causes a soft transition of molten material on the surface. A similar effect was obtained by Klocke et al. [51].

**Figure 10:**
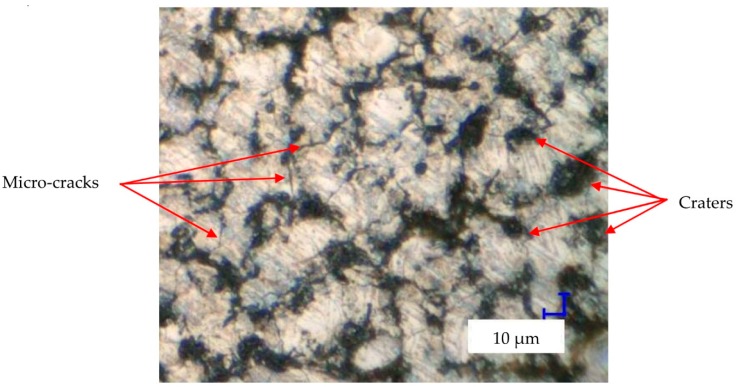
Structure of the surface after PMEMD with negative polarity RGO = 0.1% *Uc* = 25 V, *I* = 1 A, *t*_on_ = 5 µs, *t*_off_ = 5 µs.

In EDM, the discharge energy causes local melting and evaporation of material. A thin liquid metal layer that was not previously removed from the crater by the end of the discharge resolidifies on the crater. Shrinkage of the material occurs, and tensile stresses are generated. Exceeding the maximum tensile strength of the material produces microcracks. These surface defects reduce fatigue and corrosion resistance of the material. The metallographic images of the material’s surface after PMEDM with 0.1% RGO and negative polarity (Figure 9d) show no evidence of microcracks, but analyses of images showing surface topography reveal that microcracks also occur in this case (Figure 10). 

## 4. Conclusions

In this study, PMEDM with RGO of the tool steel 55NiCrMoV7 was analyzed. The effect of PMEDM on the surface roughness and integrity properties depends on the discharge current, pulse time, the polarity of the electrode, and concentration (%) of RGO in the dielectric. The results of the experiments indicate that when performing EDM with RGO, there is a dispersion of the discharges on the RGO flakes. This leads to a multiplication of the discharges during a single pulse.

Based on theoretical analyses and experimental research, the following conclusions are drawn:

- An RGO concentration above 1% in the dielectric combined with low values of discharge current and pulse time cause the dispersion of electrons on RGO flakes without effective discharges causing material removal.
- Depending on the value of the RGO concentration in the dielectric and the type of polarity used, there are significant differences in surface topography and thickness of the recast layer.
- Reduction in the discharge energy by dispersion on RGO leads to the generation of craters with a smaller diameter and depth compared with those produced by machining without RGO in the dielectric.
- PMEDM with RGO causes more stable electrical discharges with less energy and results in a uniform thickness of the recast layer.
- The best properties of surface roughness and the recast layer are obtained with 0.1% RGO in the dielectric and negative polarity.

## Figures and Tables

**Table 1:** PMEDM conditions.

Electrode	Copper
Material	tool steel 55NiCrMoV7
Polarity of electrode	positive polarity, negative polarity
Discharge current *I* (A)	1; 2
Open voltage U_0_ (V)	225 V
Discharge voltage (V)	25
pulse time *t*_on _(μs)q	5; 8
time interval *t*_off _(μs)	5
RGO in dielectric (%)	0; 0.1; 0.5; 1
Manufacturing time (s)	360

**Table 2:** The values of the machining parameters applied in the experimental design.

No.	RGO in Dielectric%	EDM Parameters	Positive Polarity				Negative Polarity			
			*Sa* (µm)	*Sdq* (µm/µm)	*Ssc* (1/µm)	*Sds* (pks/mm^2^)	*Sa* (µm)	*Sdq* (µm/µm)	*Ssc* (1/µm)	*Sds* (pks/mm^2^)
1.	0	*I* = 1 A, *t*_on_ = 5 µs	1.41	0.213	0.063	1063	1.12	0.067	0.035	1110
2.	0.1	*I* = 1 A, *t*_on_ = 5 µs	0.58	0.154	0.065	2356	0.44	0.067	0.035	1912
3.	0.5	*I* = 2 A, *t*_on_ = 8 µs	1.37	0.188	0.067	1406	1.11	0.106	0.039	952
4.	1	*I* = 2 A, *t*_on_ = 8 µs	1.56	0.213	0.066	1149	1.13	0.114	0.042	1213

## References

[1] Kunieda M., Lauwers B., Rajurkar K.P., Schumacher B.M. (2005). Advancing EDM through Fundamental Insight into the Process. CIRP Ann. Manuf. Technol..

[2] Li K., Xu G., Huang X., Xie Z., Gong F. (2019). Manufacturing of Micro-Lens Array Using Contactless Micro-Embossing with an EDM-Mold. Appl. Sci..

[3] Świercz R., Oniszczuk-Świercz D., Dabrowski L. (2018). Electrical discharge machining of difficult to cut materials. Arch. Mech. Eng..

[4] Liu Y., Chang H., Zhang W., Ma F., Sha Z., Zhang S. (2018). A Simulation Study of Debris Removal Process in Ultrasonic Vibration Assisted Electrical Discharge Machining (EDM) of Deep Holes. Micromachines.

[5] Ruszaj A., Gawlik J., Skoczypiec S. (2016). Electrochemical Machining—Special Equipment and Applications in Aircraft Industry. Manag. Prod. Eng. Rev..

[6] Skoczypiec S. (2016). Discussion of ultrashort voltage pulses electrochemical micromachining: A review. Int. J. Adv. Manuf. Technol..

[7] Dai H., Sun J., Li Z., Zhao J., Yu X., Fang H., Chen J. (2018). Fabrication of Metallic Glass Layers on Al Alloys with Improved Corrosion Resistance and Micro-Hardness by Pulsed Electrical Discharge Treatment. Appl. Sci..

[8] Rokosz K., Hryniewicz T., Matýsek D., Raaen S., Valíček J., Dudek Ł., Harničárová M. (2016). SEM, EDS and XPS Analysis of the Coatings Obtained on Titanium after Plasma Electrolytic Oxidation in Electrolytes Containing Copper Nitrate. Materials.

[9] Chmielewski T., Siwek P., Chmielewski M., Piątkowska A., Grabias A., Golański D. (2018). Structure and Selected Properties of Arc Sprayed Coatings Containing In-Situ Fabricated Fe-Al Intermetallic Phases. Metals.

[10] Hryniewicz T., Rokosz K., Rokicki R., Prima F. (2015). Nanoindentation and XPS Studies of Titanium TNZ Alloy after Electrochemical Polishing in a Magnetic Field. Materials.

[11] Gołąbczak M., Święcik R., Gołąbczak A., Nouveau C., Jacquet P., Blanc C. (2018). Investigations of surface layer temperature and morphology of hard machinable materials used in aircraft industry during abrasive electrodischarge grinding process. Mater. Und Werkst..

[12] Spadło S., Depczyński W., Młynarczyk P. (2017). Selected properties of high velocity oxy liquid fuel (HVOLF)—Sprayed nanocrystalline WC-CO INFRALLOYTM S7412 coatings modified by high energy electric pulse. Metalurgija.

[13] Salacinski T., Winiarski M., Chmielewski T., Świercz R. Surface finishing using ceramic fibre brush tools. Proceedings of the 26th International Conference on Metallurgy and Materials.

[14] Bańkowski D., Spadło S. (2017). The Aplication of Vibro—Abrasive Machining for Smoothing of Castings. Arch. Foundry Eng..

[15] Chmielewski T., Golański D., Włosiński W., Zimmerman J. (2015). Utilizing the energy of kinetic friction for the metallization of ceramics. Bull. Pol. Acad. Sci. Tech. Sci..

[16] Rudrapati R., Bandyopadhyay A., Pal P.K. (2013). Multi-objective optimization in traverse cut cylindrical grinding. Adv. Mater. Manuf. Charact..

[17] Sanchez J.A., Conde A., Arriandiaga A., Wang J., Plaza S. (2018). Unexpected Event Prediction in Wire Electrical Discharge Machining Using Deep Learning Techniques. Materials.

[18] Świercz R., Oniszczuk-Świercz D., Chmielewski T. (2019). Multi-Response Optimization of Electrical Discharge Machining Using the Desirability Function. Micromachines.

[19] Bilal A., Jahan M.P., Talamona D., Perveen A. (2019). Electro-Discharge Machining of Ceramics: A Review. Micromachines.

[20] Melnik Y.A., Kozochkin M.P., Porvatov A.N., Okunkova A.A. (2018). On Adaptive Control for Electrical Discharge Machining Using Vibroacoustic Emission. Technologies.

[21] Flaño O., Ayesta I., Izquierdo B., Sánchez J.A., Zhao Y., Kunieda M. (2018). Improvement of EDM performance in high-aspect ratio slot machining using multi-holed electrodes. Precis. Eng..

[22] Świercz R., Oniszczuk-Świercz D. (2017). Experimental Investigation of Surface Layer Properties of High Thermal Conductivity Tool Steel after Electrical Discharge Machining. Metals.

[23] Sanchez J.A., de Lacalle L.N.L., Lamikiz A. (2004). A computer-aided system for the optimization of the accuracy of the wire electro-discharge machining process. Int. J. Comput. Integr. Manuf..

[24] Sanchez J.A., Plaza S., Lacalle L.N.L.D., Lamikiz A. (2006). Computer simulation of wire-EDM taper-cutting. Int. J. Comput. Integr. Manuf..

[25] Kozak J., Rozenek M., Dabrowski L. (2003). Study of electrical discharge machining using powder-suspended working media. Proc. Inst. Mech. Eng. Part B J. Eng. Manuf..

[26] Murray J.W., Sun J., Patil D.V., Wood T.A., Clare A.T. (2016). Physical and electrical characteristics of EDM debris. J. Mater. Process. Technol..

[27] Wang X., Liu Y., Zhang Y., Sun Q., Li Z., Shen Y. (2016). Characteristics of plasma channel in powder-mixed EDM based on monopulse discharge. Int. J. Adv. Manuf. Technol..

[28] Mohanty S., Mishra A., Nanda B.K., Routara B.C. (2018). Multi-objective parametric optimization of nano powder mixed electrical discharge machining of AlSiCp using response surface methodology and particle swarm optimization. Alex. Eng. J..

[29] Öpöz T.T., Yaşar H., Ekmekci N., Ekmekci B. (2018). Particle migration and surface modification on Ti6Al4V in SiC powder mixed electrical discharge machining. J. Manuf. Process..

[30] Marashi H., Jafarlou D.M., Sarhan A.A.D., Hamdi M. (2016). State of the art in powder mixed dielectric for EDM applications. Precis. Eng..

[31] Marashi H., Sarhan A.A.D., Hamdi M. (2015). Employing Ti nano-powder dielectric to enhance surface characteristics in electrical discharge machining of AISI D2 steel. Appl. Surf. Sci..

[32] Nguyen T.D., Nguyen P.H., Banh L.T. (2019). Die steel surface layer quality improvement in titanium μ-powder mixed die sinking electrical discharge machining. Int. J. Adv. Manuf. Technol..

[33] Amorim F.L., Dalcin V.A., Soares P., Mendes L.A. (2017). Surface modification of tool steel by electrical discharge machining with molybdenum powder mixed in dielectric fluid. Int. J. Adv. Manuf. Technol..

[34] Toshimitsu R., Okada A., Kitada R., Okamoto Y. (2016). Improvement in Surface Characteristics by EDM with Chromium Powder Mixed Fluid. Procedia CIRP.

[35] Shard A., Shikha D., Gupta V., Garg M.P. (2018). Effect of B4C abrasive mixed into dielectric fluid on electrical discharge machining. J. Braz. Soc. Mech. Sci. Eng..

[36] Kumar A., Mandal A., Dixit A.R., Das A.K. (2018). Performance evaluation of Al_2_O_3_ nano powder mixed dielectric for electric discharge machining of Inconel 825. Mater. Manuf. Process..

[37] Patel S., Thesiya D., Rajurkar A. (2018). Aluminium powder mixed rotary electric discharge machining (PMEDM) on Inconel 718. Aust. J. Mech. Eng..

[38] Talla G., Gangopadhyay S., Biswas C.K. (2017). Influence of graphite powder mixed EDM on the surface integrity characteristics of Inconel 625. Part. Sci. Technol..

[39] Mohanty S., Kumar V., Kumar Das A., Dixit A.R. (2019). Surface modification of Ti-alloy by micro-electrical discharge process using tungsten disulphide powder suspension. J. Manuf. Process..

[40] Al-Khazraji A., Amin S.A., Ali S.M. (2016). The effect of SiC powder mixing electrical discharge machining on white layer thickness, heat flux and fatigue life of AISI D2 die steel. Eng. Sci. Technol. Int. J..

[41] Hourmand M., Sarhan A.A.D., Farahany S., Sayuti M. (2018). Microstructure characterization and maximization of the material removal rate in nano-powder mixed EDM of Al-Mg_2_Si metal matrix composite—ANFIS and RSM approaches. Int. J. Adv. Manuf. Technol..

[42] Bains P.S., Sidhu S.S., Payal H.S., Kaur S. (2019). Magnetic Field Influence on Surface Modifications in Powder Mixed EDM. Silicon.

[43] Rani J.R., Thangavel R., Oh S.-I., Lee Y.S., Jang J.-H. (2019). An Ultra-High-Energy Density Supercapacitor; Fabrication Based on Thiol-functionalized Graphene Oxide Scrolls. Nanomaterials.

[44] Yang C.-R., Tseng S.-F., Chen Y.-T. (2018). Characteristics of Graphene Oxide Films Reduced by Using an Atmospheric Plasma System. Nanomaterials.

[45] Bernal M.M., Tortello M., Colonna S., Saracco G., Fina A. (2017). Thermally and Electrically Conductive Nanopapers from Reduced Graphene Oxide: Effect of Nanoflakes Thermal Annealing on the Film Structure and Properties. Nanomaterials.

[46] Stylianakis M.M., Viskadouros G., Polyzoidis C., Veisakis G., Kenanakis G., Kornilios N., Petridis K., Kymakis E. (2019). Updating the Role of Reduced Graphene Oxide Ink on Field Emission Devices in Synergy with Charge Transfer Materials. Nanomaterials.

[47] Pisarek M., Holdynski M., Krawczyk M., Nowakowski R., Roguska A., Malolepszy A., Stobinski L., Jablonski A. (2016). Surface characterization of graphene based materials. Appl. Surf. Sci..

[48] Stobinski L., Lesiak B., Malolepszy A., Mazurkiewicz M., Mierzwa B., Zemek J., Jiricek P., Bieloshapka I. (2014). Graphene oxide and reduced graphene oxide studied by the XRD, TEM and electron spectroscopy methods. J. Electron Spectrosc. Relat. Phenom..

[49] Jahan M.P., Rahman M., Wong Y.S. (2011). Study on the nano-powder-mixed sinking and milling micro-EDM of WC-Co. Int. J. Adv. Manuf. Technol..

[50] Chu X., Zhu K., Wang C., Hu Z., Zhang Y. (2016). A Study on Plasma Channel Expansion in Micro-EDM. Mater. Manuf. Process..

[51] Klocke F., Lung D., Antonoglou G., Thomaidis D. (2004). The effects of powder suspended dielectrics on the thermal influenced zone by electrodischarge machining with small discharge energies. J. Mater. Process. Tech..

